# Giving information strategically and transparently: A pilot trial of the Oncolo‐GIST intervention to promote patients' prognostic understanding

**DOI:** 10.1002/cam4.6420

**Published:** 2023-08-08

**Authors:** Holly G. Prigerson, David Russell, Sophia E. Kakarala, Heather M. Derry‐Vick, Manish A. Shah, Ashish Saxena, Valerie F. Reyna, Allyson Ocean, Ronald Scheff, Paul K. Maciejewski, Andrew S. Epstein

**Affiliations:** ^1^ Department of Medicine, Division of Geriatrics and Palliative Medicine Weill Cornell Medicine New York New York USA; ^2^ Cornell Center for Research on End‐of‐Life Care Weill Cornell Medicine New York New York USA; ^3^ Department of Sociology Appalachian State University Boone North Carolina USA; ^4^ Hackensack Meridian School of Medicine New Jersey USA; ^5^ Department of Medicine, Division of Hematology and Medical Oncology Weill Cornell Medicine New York New York USA; ^6^ Cornell University, Human Neuroscience Institute Ithaca New York USA; ^7^ Department of Radiology Weill Cornell Medicine New York New York USA; ^8^ Memorial Sloan Kettering Cancer Center New York New York USA

**Keywords:** cancer, communication intervention, prognostic understanding, terminal illness acknowledgment

## Abstract

**Purpose:**

Most patients with cancer lack the prognostic understanding necessary to make informed decisions. We tested the feasibility and acceptability of the Oncolo‐GIST (“Giving Information Strategically and Transparently, GIST”) intervention and explored its associations with patients' improved prognostic understanding.

**Methods:**

The Oncolo‐GIST intervention distills prognostic discussions into easy‐to‐understand talking points. Patients with metastatic cancers that progressed on ≥1 line of chemotherapy and not expected to survive 12 months (*n* = 31) were recruited from October 2020 through November 2022. We compared patients who discussed their progressive scans with an oncologist trained in the GIST technique or not (i.e., usual care). A primary outcome was prognostic understanding (e.g., patients reporting a life‐expectancy of months) assessed within a week of the scan discussion visit.

**Results:**

Oncologists (*n* = 4) appeared receptive to the Oncolo‐GIST intervention and scored nearly perfectly on post‐training tests of material mastery after a < 2‐h tutorial. Post‐scan discussion visit, 100% of patients who met with an Oncolo‐GIST‐trained clinician understood that their cancer was considered incurable (a 31% improvement from pre‐visit) compared with 91% of patients meeting with usual care oncologists (an 18% improvement); 33% of patients who met with an Oncolo‐GIST‐trained oncologist understood that they likely had months, not years, compared to 18% in the usual care group. No statistically significant differences emerged for these changes, nor for therapeutic alliance, anxiety, or depression scores between groups.

**Conclusion:**

Oncolo‐GIST appears to be an easily learned approach to improve prognostic understanding that neither undermines therapeutic alliances nor increases patients' anxiety or depressive symptoms. Efficacy testing in a larger trial is warranted.

## Abstract 2

Most patients with advanced cancer do not realize how close to death they are. The Oncolo‐GIST communication intervention is easy for physicians to learn and appears to promote patient prognostic understanding while not harming the doctor‐patient relationship nor patient mental health.

## 1 INTRODUCTION

Despite the recent advances in cancer treatments, the disease often does progress, and patients can then reliably be expected to have months, not years, left to live. We have shown that oncologists can accurately predict when patients have a life expectancy of 12 months or less,^1^, ^2^ and that their prognostic estimates are most accurate (an average 0.2 month underestimate) when their patients are within 9–12 months of death.^2^ By contrast, we found that only 5% of patients within a median of 5 months of death accurately understood that their oncologists considered them to be terminally ill, at a late‐/end‐stage of their disease, to have incurable cancer, and that they likely had months, not years, left to live.^3^ Evidently, a large majority of patients with cancer whom we have studied lacked the prognostic understanding necessary to make informed choices about their end‐of‐life care.

Patients who grasp that they are dying, (e.g., the 5% who “got the gist” of their prognosis),^3^ tend to have higher rates of advance care planning (ACP),^1^ receive less burdensome and unbeneficial care (e.g., cardiopulmonary resuscitation and intensive care),^1^, ^4^, ^5^ and receive more value‐consistent care.^4^ We have found that patient prognostic understanding is improved by oncologist discussions of life‐expectancy.^2^, ^3^, ^6^, ^7^ Nevertheless, despite 71% of patients wanting to discuss prognosis with their oncologists (83% of young adult patients with cancer thought prognostic information was extremely/very important),^8^ only 18% of patients with cancer within months of death reported that they had discussed prognosis with their oncologist.^2^ Not only do oncologists appear to discuss prognosis less than patients want,^2^ but even when prognostic discussions do occur, approaches that are more matter‐of‐fact than vague have been shown to be more effective in promoting patients' prognostic understanding.^6^ Thus, research indicates a need to improve how oncologists communicate to promote patients' prognostic understanding.

To address this need we developed the “Giving Information Simply and Transparently” (GIST) communication technique for oncologists, which we call the Oncolo‐GIST intervention.^9^ This streamlined approach to prognostic discussions is designed to be an easy‐to‐learn and effective way to promote patients' prognostic understanding. Compared to traditional approaches which emphasize numerical or medical details (e.g., tumor size, rate of growth), Oncolo‐GIST is based on Reyna's Fuzzy‐trace theory of decision‐making,^10^, ^11^ emphasizing the need for an understanding of the bottom‐line gist of treatment options. The Oncolo‐GIST approach distills prognostic discussions to communicate end‐of‐life decision‐making essentials (e.g., that a patient's cancer is considered incurable, and that they likely have months, not years, to live).

The present study reports on a trial of Oncolo‐GIST for patients with metastatic cancers that progressed on at least 1 line of chemotherapy and whom oncologists did not expect to survive 12 months. Patient assessments occurred after enrollment and again within 1 week after the clinic visit in which progressive scan results were discussed. We sought to determine if patients who met with an oncologist trained in Oncolo‐GIST were more likely than patients who received usual care to report (a) that their cancer was considered by oncologists to be incurable; (b) that they likely have “months to live,” and (c) are at a late‐/end‐stage of their illness; that is, that they “got the gist” of their poor prognosis.

## 2 METHODS

This trial was funded by NIH/NINR (NR018693: MPIs: Prigerson/Epstein) to determine if the Oncolo‐GIST intervention could improve patients' prognostic understanding. Enrollment of the first trial participant occurred on October 25, 2020, and the study primary completion date was November 8, 2022. This study was approved by the Weill Cornell Medicine Institutional Review Board.

### 2.1 The Oncolo‐GIST technique

The premise of GIST is not merely to use simple words and short phrases, but to communicate in a way to maximize the likelihood that patients grasp essential medical facts relevant to the healthcare choices before them. Oncolo‐GIST training focused on the following four steps:

- Step #1: GIVING (**G**) the scan result using simple language such as “the scan showed the cancer is growing, which means that treatment is not stopping the spread of cancer. This means that your condition is worsening, and now more serious.”
- Step #2: INFORMING (**I**) prognosis by using language such as “I wish it were not the case, but for people with your prognosis, when the cancer progresses on treatment, the average life‐expectancy is months, not years, to live. This means that for almost all patients in your situation, I expect survival from now to be months, but not years.”
- Step #3: STRATEGIZING (**S**) next steps using language such as “hoping for the best and preparing for the worst,” and then making a medical recommendation such as “Given what I know about your cancer, the benefits and harms of various treatments, and what's important to you, I recommend we…”.
- Step #4: TRANSPARENTLY (**T**) asking the patient what was heard by using language such as “Help me to understand what you've taken away from our discussion. Please tell me in your own words what the scan results mean… and I will fill in any gaps.”

### 2.2 Oncologist participation

Recruitment of Oncolo‐GIST physicians used a convenience sample of oncologists on the GI and thoracic services at Weill Cornell Medicine/New York Presbyterian Hospital. Each oncologist who was approached agreed to participate in the trial, suggesting receptivity to the technique.

Participating oncologists either received the Oncolo‐GIST Version 2.0.^9^ training or not (i.e., usual care). In Phase 1 of this project, we had obtained feedback from relevant stakeholders (e.g., oncologists, palliative care clinicians, caregivers of patients who had died) on the Oncolo‐GIST approach. Recommendations were reviewed by the study team and incorporated into Version 2.0,^9^ which was the version used to train those in the intervention arm.

Study MPIs (ASE, HGP) led the training, which included a < 2‐h session in which the material for communicating key prognostic talking points was presented, brief video demonstrations were reviewed, and a role‐play undertaken. Questions regarding the technique were answered at that time and following training, the oncologist trainees were tested with a brief survey of the four Oncolo‐GIST talking points and communication recommendations in the Oncolo‐GIST manual.

One of the three trained oncologists was removed from study participation prior to patient enrollment due to non‐adherence to the Oncolo‐GIST manual guidelines (i.e., this oncologist refused to communicate using the Oncolo‐GIST approach). This left two trained and two untrained oncologists participating in this trial. The two trained oncologists completed a posttraining test and were found to have nearly perfect mastery of the material (e.g., >95% correct responses), suggesting that it was easy to learn. We then proceeded to pilot test Oncolo‐GIST 2.0.

### 2.3 Patient participation

#### 2.3.1 Patient eligibility criteria

Patients were included if they were 18 years of age or older, receiving care including regular scans and ≥2 visits with the referring oncologist, had a radiologist scan impression revealing progressive disease on ≥1 line of systemic cancer therapy, had a life‐expectancy of ≤12 months based on their oncologists' opinion, and who were fluent in English (e.g., excluding patients if their medical chart indicated that they needed an interpreter). Patients who scored <6 on the short portable mental status questionnaire^12^ were excluded due to cognitive impairment, and those scoring below a seventh grade reading level on the Rapid Estimate of Adult Literacy in Medicine (REALM)^13^ were excluded due to insufficient health literacy. Informed consent for all study participants occurred in‐person or remotely via phone call while using a secure REDCap‐based electronic consent system.

Eligible patients were randomly assigned to meet with either a GIST‐trained oncologist or an oncologist who did not receive such training. Patients were assessed by trained research staff at a baseline evaluation, which occurred after consent, and then again at follow‐up within 1 week of the clinic visit in which progressive scan results were discussed. When a patient's first scan on the study did not show disease progression, study staff instead contacted them for follow‐up at a subsequent scan (within 1 year) that did show progression.

### 2.4 Study measures

#### 2.4.1 Patient characteristics

Patients' age, sex, race/ethnicity, level of educational attainment, religion, spirituality, and primary cancer diagnosis (i.e., gastrointestinal cancer or thoracic cancer) were assessed at baseline via self‐report and verified against information found in their medical record.

#### 2.4.2 Primary outcomes

##### Patient prognostic understanding

Patient prognostic understanding was assessed with our validated measure of prognostic understanding^3^ administered at the baseline assessment and again at follow‐up within a week of the post‐scan visit. The assessment included three items: (1) patients' recognition that their cancer was considered to be incurable, (2) acknowledgment of the advanced stage (i.e., late/end stage) of their disease, and (3) expectation to live months as opposed to years. Responses were coded 1 to indicate the correct understanding of each of these elements (otherwise scores were 0). These three indicators were then added together to produce a summary score of prognostic understanding (possible range, 0–3). Differences between pre‐ and post‐scan visit (baseline and T1, respectively) prognostic understanding scores were used to define changes in prognostic understanding by a patient between the pre‐ and post‐scan visit interviews.

#### 2.4.3 Secondary outcomes

##### Therapeutic alliance

Therapeutic alliance was assessed using our validated Human Connection scale.^14^ This assessment is an application of the notion in psychotherapy emphasizing the importance of the therapeutic bond to treatment efficacy^15^ and applied to the bond between patients with cancer and their oncologists. It assesses the five key elements of the therapeutic alliance—the extent to which the patient feels (1) that the oncologist listens to and understands the patient's concerns about their illness, (2) that the relationship involves mutual caring and respect, (3) that the patient understands the information being shared by the oncologist, (4) that the patient trusts the oncologist, and (5) that the oncologist and patient work well together.^14^

##### Depression and anxiety

Depression and anxiety were assessed with the depression and anxiety subscales of the validated hospital anxiety and depression scale (HADS), which measures depression and anxiety symptom severity.^16^ We do not present results from other assessments (e.g., quality of life) as they were less central and had missing data.

### 2.5 Statistical analysis

Descriptive statistics were used to characterize oncologists' mastery of the training material and participating patients' baseline characteristics. Two‐sample *t*‐tests were used to determine if the Oncolo‐GIST and usual care groups differed to a significant degree (*p* < 0.05) from each other on mean overall prognostic understanding scores. Likelihood ratio tests were used to determine if the Oncolo‐GIST and usual care groups differed to a significant degree (*p* < 0.05) from each other with respect to baseline and post‐scan assessment changes in categorical outcomes (e.g., improved/stable/decreased prognostic understanding).

## 3 RESULTS

**FIGURE 1:**
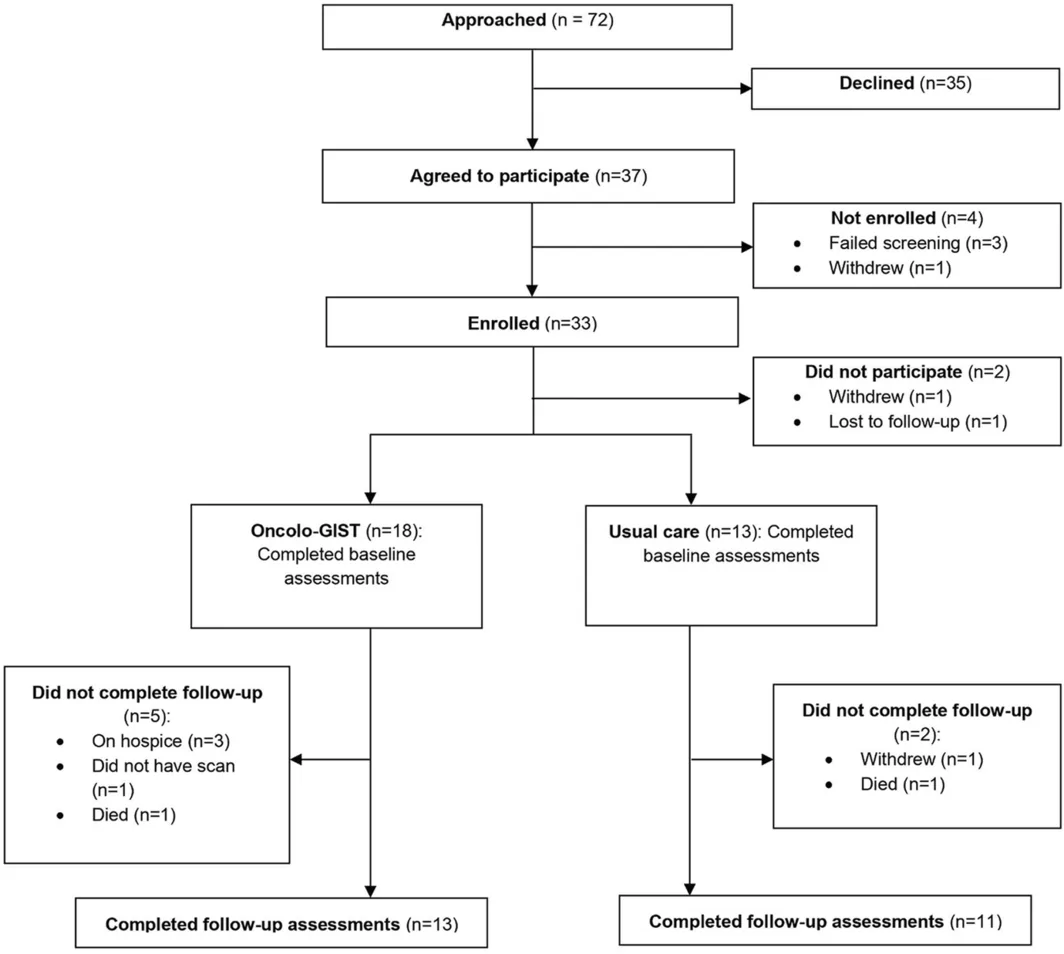
CONSORT diagram of study recruitment and enrollment.

Screening and recruitment for this study are detailed in the CONSORT diagram below (Figure 1). 73 patients were approached for trial participation; 37 agreed and were then screened, yielding a 51% participation rate. Of these 37, 2 did not pass the cognitive screen, 1 did not pass the health literacy screen, and 1 opted out of study participation, yielding 33 enrolled study participants. Of these, 31 patients completed the baseline assessment (1 person withdrew before completing the assessment). A total of 24 study participants completed the follow‐up (post‐scan) surveys (77% retention): 4 patients never had a scan revealing progressive illness prior to close of the study, 1 patient withdrew citing time constraints, 2 patients died before completing their follow‐up surveys.

As displayed in Table 1, the baseline sample (*N* = 31) was comprised of 18 patients with cancer who met with an Oncolo‐GIST‐trained oncologist and 13 patients with cancer who met with an oncologist without this training. The average age was 66 years; 65% of the patients self‐identified as male, 71% White, 13% African American/Black, 10% Hispanic, and 3% Asian; 61% were Christian; nearly 70% reported being moderately to very spiritual; and over 70% were diagnosed with a GI cancer. Oncolo‐GIST and usual care patient trial participants did not differ significantly (*p* < 0.05) on these characteristics.

**TABLE 1:** Patient characteristics by study group.

	All patients (*N* = 31) mean (SD) or % (*n*)	Oncolo‐GIST arm (*n* = 18) mean (SD) or % (*n*)	Usual care arm (*n* = 13) mean (SD) or % (*n*)	*p‐*value
Age mean (Standard Deviation)	66.3 (12.4)	69.7 (12.6)	61.5 (10.9)	0.065
Sex				
Male	64.5% (20)	55.6% (10)	76.9% (10)	0.22
Female	35.5% (11)	44.4% (8)	23.1% (3)	
Race/ethnicity				
White non‐Hispanic	71.0% (22)	72.2% (13)	69.2% (9)	0.502
Black non‐Hispanic	12.9% (4)	16.7% (3)	7.7% (1)	
Hispanic	9.7% (3)	5.6% (1)	15.4% (2)	
Asian non‐Hispanic	3.2% (1)	0.0% (0)	7.7% (1)	
Missing	3.2% (1)	5.6% (1)	0.0% (0)	
Education				
Less than high school	3.2% (1)	0.0% (0)	7.7% (1)	0.219
High school	16.1% (5)	27.8% (5)	0.0% (0)	
Some college	16.1% (5)	16.7% (3)	15.4% (2)	
College degree	29.0% (9)	22.2% (4)	38.5% (5)	
Graduate degree	35.5% (11)	33.3% (6)	38.5% (5)	
Religion				
Christian	61.3% (19)	66.7% (12)	53.8% (7)	0.103
Jewish	12.9% (4)	5.6% (1)	23.1% (3)	
Muslim	6.5% (2)	5.6% (1)	7.7% (1)	
Other religion	6.5% (2)	0.0% (0)	15.4% (2)	
No religion	12.9% (4)	22.2% (4)	0.0% (0)	
Spirituality				
Very spiritual	32.3% (10)	33.3% (6)	30.8% (4)	0.612
Moderately spiritual	35.5% (11)	38.9% (7)	30.8% (4)	
A little spiritual	12.9% (4)	16.7% (3)	7.7% (1)	
Not at all spiritual	16,1% (5)	11.1% (2)	23.1% (3)	
Do not know	3.2% (1)	0.0% (0)	7.7% (1)	
Cancer type				
Gastrointestinal	71.0% (22)	61.1% (11)	84.6% (11)	0.155
Thoracic	29.0% (9)	38.9% (7)	15.4% (2)	

*Note*: *p*‐values reflect the results of *t‐*tests for comparison of means (for quantitative variables) between the Oncolo‐GIST and usual care groups and chi‐squared tests for comparison of nominal (categorical) variables.

The results in Table 2 indicate that prognostic understanding did not differ significantly between study groups at baseline. Prognostic understanding at baseline was highest for the item that their cancer was considered incurable (69% and 73% of patients in the Oncolo‐GIST and usual care groups, respectively), followed by understanding that their cancers were at a late/end stage (50% and 64%), and lowest for understanding that their life expectancy was months (17% and 9%). When assessed again at post‐scan, 31% of patients in the Oncolo‐GIST group improved in their understanding that their cancer was not expected to be cured (i.e., 4 of 13 patients expressed understanding at post‐scan that they did not communicate at baseline), compared with 18% of patients in the usual care group (i.e., 2 of 11 patients expressed understanding not communicated at baseline). Further, 25% of patients (3 of 12) in the Oncolo‐GIST group had improved late/end stage understanding at post‐scan compared with 18% of patients (2 of 11) in usual care. Notably, 17% (2 of 12) of patients in the Oncolo‐GIST group decreased in their understanding versus 27% of patients (3 of 11) who spoke with a usual care oncologist. Lastly, 25% of patients (3 of 12) in the Oncolo‐GIST group improved in their understanding that their life expectancy is months compared with 18% (2 of 11) in usual care.

**TABLE 2:** Changes in prognostic understanding: Baseline to post‐scan interviews by study arm.

	Oncolo‐GIST; *n* = 13		Usual care; *n* = 11		*p*‐value
	Mean (SD) or % (*n*)	*n*	Mean (SD) or % (*n*)	*n*	
Prognostic understanding					
My cancer cannot be cured (baseline; % understanding)	69.2% (9)	13	72.7% (8)	11	
My cancer cannot be cured (post‐scan; % understanding)	100% (13)	13	90.9% (10)	11	
My cancer cannot be cured (change between baseline and post‐Scan)					
Improved understanding	30.8% (4)	13	18.2% (2)	11	0.474
Stable	69.2% (9)		81.8% (9)		
Decreased understanding	0.0% (0)		0% (0)		
Late/end stage of cancer (baseline; % understanding)	50.0% (6)	12	63.6% (7)	11	
Late/end stage of cancer (post‐scan; % understanding)	58.3% (7)	12	54.5% (6)	11	
Late/end stage of cancer (change between baseline and post‐scan)					
Improved understanding	25.0% (3)	12	18.2% (2)	11	0.804
Stable	58.3% (7)		54.5% (6)		
Decreased understanding	16.7% (2)		27.3% (3)		
Life expectancy is months (baseline)	16.7% (2)	12	9.1% (1)	11	
Life expectancy is months (post‐scan)	33.3% (4)	12	18.2% (2)	11	
Life expectancy is months (change between baseline and post‐Scan)					
Improved understanding	25.0% (3)	12	18.2% (2)	11	0.924
Stable	66.7% (8)		72.7% (8)		
Decreased understanding	8.3% (1)		9.1% (1)		
Change in prognostic understanding (sum 3‐items) between baseline and post‐Scan	0.58 (1.00)	12	0.18 (1.25)	11	0.402
+3 points	0.0% (0)		9.1% (1)		
+2 points	16.7% (2)		0.0% (0)		
+1 points	41.7% (5)		27.3% (3)		
No change	25.0% (3)		27.3% (3)		
‐1 points	16.7% (2)		36.4% (4)		

*Note*: *p*‐values for changes in the categorical outcome variables (improved/stable/decreased understanding between baseline and post‐scan interviews) between study groups are for likelihood ratio tests; the *p‐*value for changes in the mean overall prognostic understanding score (sum of three items) between study groups reflects the results from a two‐sample *t*‐test. This table includes the sample with complete follow‐up data (*n* = 24).

**FIGURE 2:**
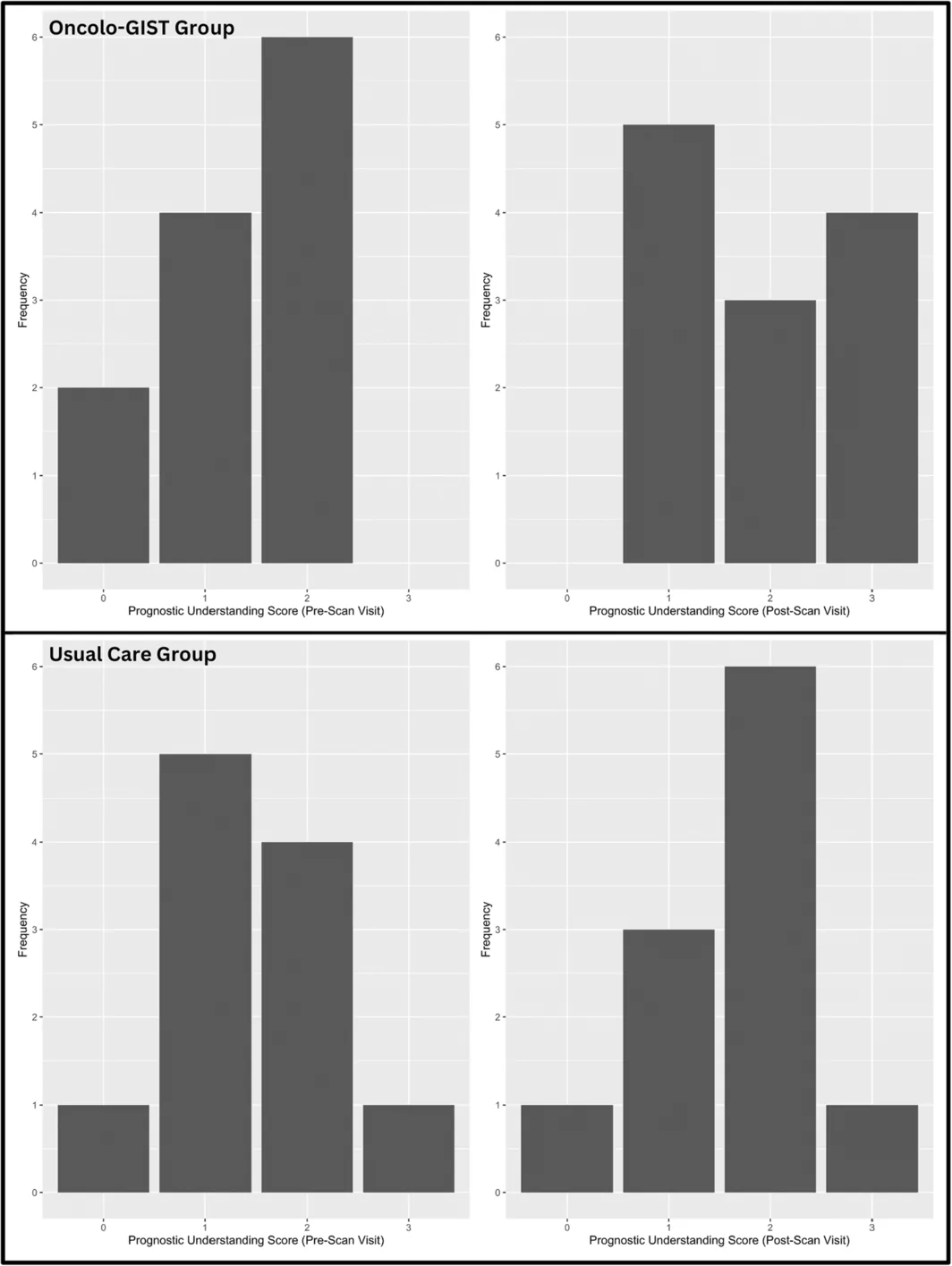
Histograms showing distributions of prognostic understanding scores at pre‐ and post‐scan visits.

The overall change in prognostic understanding based on a summary score from these three items was positive for both groups, but the mean improvement was slightly higher (Mean = 0.58; Standard Deviation = 1.00) for the Oncolo‐GIST group compared to the usual care group (Mean = 0.18; Standard Deviation = 1.25); changes in overall prognostic understanding are displayed in Figure 2. Changes in overall prognostic understanding between study groups did not achieve a level of statistical significance.

**FIGURE 3:**
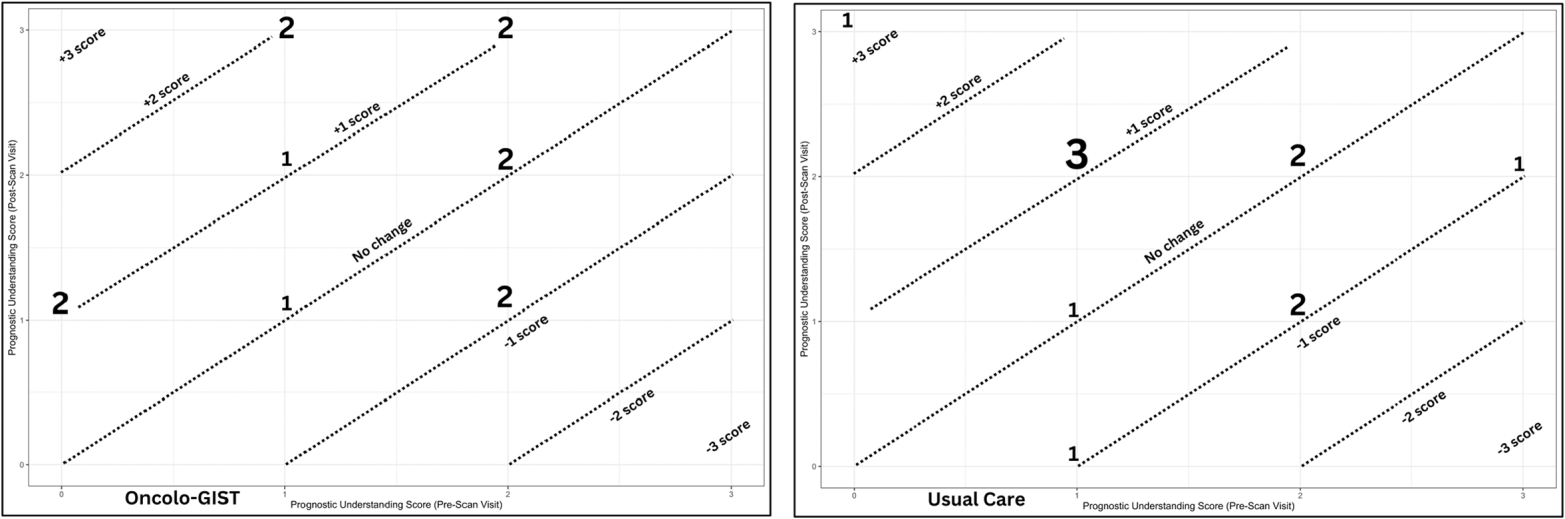
Weighted scatter plots showing changes in prognostic understanding scores between pre‐ and post‐scan visits.

To display these results another way, Figure 3 presents weighted scatter plots showing the joint distribution of pre‐ and post‐scan overall prognostic understanding scores for each study group. These plots show 45‐degree lines corresponding to changes in prognostic understanding between pre‐ and post‐scan assessments (e.g., an improvement of +2 points or − 1 points), as well as the number of patients with each combination of scores weighted by frequency. These graphs reveal that 7 of 12 patients (58%) in the Oncolo‐GIST group improved in their overall prognostic understanding by +1 points or greater compared with 4 of 11 (36%) in the usual care group.

In Table 3 we see that there were no statistically significant differences in changes in therapeutic alliance, nor differences in changes in anxiety or depression, between patients who met with an Oncolo‐GIST‐trained oncologist and those whose oncologist who did not receive such training. There was no decline in either group in liking, trusting, or feeling like their oncologist was honest. The Human Connection scale 3‐item summary scores, on average, improved slightly (0.08) among patients who met with an Oncolo‐GIST‐trained oncologist and a bit more (0.27) among patients meeting with an oncologist in the usual care group. While not statistically significant, patients in the Oncolo‐GIST group observed a greater decrease than patients in the usual care group on items that their doctor asked how their family was coping with their illness and were less comfortable asking questions of their physician. Additionally, patients in the usual care group had slightly greater increases in depressive symptoms (Mean = 2.45; Standard Deviation = 3.14) between baseline and post‐scan follow‐up than those in the Oncolo‐GIST group (Mean = 1.64; Standard Deviation = 3.8; *p* = 0.588).

**TABLE 3:** Change in the human connection (THC) and hospital anxiety and depression scale items between study groups.

	Oncolo‐GIST arm mean (SD)	*n*	Usual care arm mean (SD)	*n*	*p*‐value
T1‐BL Change in The Human Connection (THC) Scale [11 items]	−1.00 (1.63)	7	0.14 (1.57)	7	0.207
T1‐BL Change in The Human Connection (THC) Scale [3 items]	0.08 (1.11)	13	0.27 (1.10)	11	0.671
T1‐BL Change in The Human Connection (THC) Scale Item Oncologist takes the time to listen to concerns	−0.08 (0.64)	13	0.00 (0.45)	11	0.741
Oncologist sees patient you as whole person	−0.08 (0.28)	13	0.00 (0.00)	10	0.392
Likes oncologist	0.00 (0.00)	12	0.00 (0.00)	12	NA*
Trusts oncologist	0.00 (0.00)	12	0.00 (0.00)	11	NA*
Oncologist cares about you	−0.08 (0.49)	11	0.00 (0.00)	11	0.612
Oncologist is honest with you	0.00 (0.47)	9	0.00 (0.00)	10	1.000
Oncologist offers hope	−0.22 (0.67)	9	0.11 (0.33)	9	0.198
Oncologist is concerned about quality of life	0.23 (0.44)	13	0.27 (1.01)	11	0.893
Doctor asks how you are coping with cancer	0.15 (0.80)	13	0.09 (1.14)	11	0.875
Doctor asks how family members are coping with your illness	0.50 (1.27)	10	0.20 (0.63)	10	0.512
Feels comfortable asking doctor questions	−0.42 (0.90)	11	0.00 (0.00)	11	0.141
T1‐BL Change in HADS anxiety scale	0.64 (2.50)	11	0.40 (1.43)	10	0.796
T1‐BL Change in HADS depression scale	1.64 (3.80)	11	2.45 (3.14)	11	0.588

*Note*: *p‐*values for changes in THC and HADS anxiety and depression measures between study groups reflect two‐sample *t*‐tests. This table includes the sample with complete follow‐up data (*n* = 24).

* Unable to calculate *p*‐value due to no variation in study outcome between assessment points.

## 4 DISCUSSION

Results of this pilot of the Oncolo‐GIST technique demonstrated receptivity to the approach and improvement in patient prognostic understanding without significant harm to the patient‐oncologist relationship or to the patient's mental health. Specifically, we found that oncologists approached for this study agreed to participate, though 1 declined to adhere to its guidelines. Those who participated in the Oncolo‐GIST training demonstrated nearly perfect mastery of the Oncolo‐GIST material. Retention of patients was also good (>75%) with most participating in the follow‐up assessment. Patients who met with Oncolo‐GIST‐trained oncologists scored higher on measures of understanding that their cancer was incurable, that they were at a late/end stage of their illness, and that they likely had months, not years, left to live. Though these differences were not statistically significant, the feasibility and acceptability data along with these promising patterns suggest that testing the intervention in a larger, well‐powered trial is warranted. The approach did not appear to adversely affect the patients' relationships with their oncologists, nor did it significantly increase their symptoms of depression or anxiety. These findings suggest that the Oncolo‐GIST communication intervention is feasible and acceptable to implement in a clinical setting. Results also suggest that Oncolo‐GIST may improve patient prognostic understanding, while not appearing to harm the doctor‐patient relationship or patient mental health.

We found that at baseline, roughly 30% of enrolled patients did not realize that their oncologists considered their cancer to be incurable. This finding is consistent with what Weeks et al.^17^ revealed nearly a decade ago: that substantial proportions of lung and GI advanced patient with cancers mistakenly believe that their anti‐cancer treatments are curative. In our study, both groups improved in recognizing their cancer's incurability after the scan visit, but 100% of patients meeting with an Oncolo‐GIST‐trained oncologist understood that their cancer was not considered curable, suggesting that the intervention was effective in this regard. This is important because patients need to understand whether their cancer treatments are intended to cure them or not to make informed decisions about receiving further anti‐cancer therapy.

Over half of enrolled patients understood that their cancer was at a late/end stage at baseline. While 25% of patients who met with an Oncolo‐GIST‐trained oncologist improved in their understanding of this information after discussing progressive scan results with their oncologist, 18% of patients improved in this understanding in the usual care group. Conversely, 17% of patients in the Oncolo‐GIST arm decreased in understanding that they were at a late‐ or end‐ stage of their illness, compared to 27% in the usual care group. These results suggest that progressive scan visits do not necessarily promote patient acknowledgment of disease stage. While the Oncolo‐GIST intervention appeared to encourage greater understanding among some patients, further honing of this message may be needed to improve other patients' recognition that they are likely in the final stage of their illness.

That said, the message in greatest need of improvement appeared to be that of life expectancy. Consistent with prior research,^3^ very few patients with advanced cancers in this study realized they were within months of death (17% of the Oncolo‐GIST and 9% of the usual care patients at baseline). While both groups roughly doubled in their understanding of this information at the post‐scan follow‐up visit, 33% of patients in the Oncolo‐GIST group got the “gist,” compared to only 18% of patients in the usual care group. This is important because patients who “get the gist” that they likely have months to live tend to have higher rates of ACP,^1^ are less likely to receive burdensome, unbeneficial care,^1^, ^4^, ^5^ and are more likely to receive value‐consistent care,^4^ compared to those who do not get the gist. This underscores both the potential for interventions such as Oncolo‐GIST to improve patient prognostic understanding and the substantial room left for improvement on this target.

While efficacy testing in a larger trial is needed to draw conclusions about the significance of the effects observed in this pilot study, these preliminary findings suggest that the Oncolo‐GIST intervention may improve patient prognostic understanding and harm neither the therapeutic bond between oncologist and patient nor patient mental health. Although patients in the Oncolo‐GIST group responded more than twice as frequently than those in the usual care group that their doctor asked how their family was coping with their illness, they also reported being less comfortable asking questions of their physician (possibly because they did not want to hear more bad news). Still, overall, these results suggest that this approach appears to be a net positive for promoting informed end‐of‐life decision‐making among patients with advanced cancer.

### 4.1 Caveats

This trial was conducted at a single site and included data from only 4 oncologist participants and 31 patients with advanced cancers in their clinics. Results should, therefore, be considered cautiously and warrant confirmation in larger, multi‐site, and adequately powered studies. Recruitment coincided with the COVID‐19 pandemic, which posed several challenges to study implementation. Among these challenges were difficulties with patient recruitment and evaluation of oncologist adherence to the Oncolo‐GIST protocol. Oncologists also shared that it was difficult for them to identify eligible patients, with many dying or opting for hospice before referral. This suggests another potential target for interventions to improve prognostic understanding; that is, an identified need to improve oncologists' acknowledgment and acceptance of their patients' impending deaths. In an examination of follow‐up data we found that 73% (*n* = 24) of the patients originally recruited to the study had died within a year of their enrollment–a rate similar to the 75% accurate year‐long survival prediction among oncologists that we found in a prior observational Study.^1^ This high proportion suggests that oncologists are accurate in identifying patients who are approaching the end of life. Roughly half of patients who were approached for this trial declined study participation, which suggests that future trials should improve acceptability of the study (possibly by reducing survey burden or duration of study participation as concern about surviving to follow‐ups was expressed by some patients) to ensure more representative sampling of patient groups with the targeted poor prognosis. However, retention was adequate (24/31, 77%) among those who agreed to participate, suggesting that the procedures were feasible among those who ultimately enrolled.

## 5 CONCLUSION

Oncolo‐GIST is a potentially promising, theory‐driven, and easily learned approach to improve prognostic understanding that neither appears to undermine patients' therapeutic alliances with their oncologists nor increase their symptoms of anxiety or depression. Confirmation of effects in a larger trial is warranted.

## AUTHOR CONTRIBUTIONS

**Holly G Prigerson:** Conceptualization (lead); data curation (lead); formal analysis (equal); funding acquisition (lead); investigation (lead); methodology (lead); resources (lead); software (supporting); supervision (lead); validation (lead); visualization (supporting); writing – original draft (lead); writing – review and editing (lead). **David Russell:** Conceptualization (supporting); data curation (supporting); formal analysis (lead); funding acquisition (supporting); investigation (supporting); methodology (equal); project administration (supporting); resources (supporting); software (lead); supervision (supporting); validation (equal); visualization (equal); writing – original draft (supporting); writing – review and editing (supporting). **Sophia E. Kakarala:** Conceptualization (supporting); data curation (supporting); formal analysis (supporting); funding acquisition (supporting); investigation (supporting); methodology (supporting); project administration (lead); software (supporting); supervision (supporting); validation (supporting); visualization (supporting); writing – original draft (supporting); writing – review and editing (supporting). **Heather M. DerryVick:** Conceptualization (supporting); data curation (supporting); formal analysis (supporting); funding acquisition (supporting); investigation (supporting); methodology (supporting); project administration (supporting); software (supporting); supervision (supporting); validation (supporting); visualization (supporting); writing – original draft (supporting); writing – review and editing (supporting). **Manish A. Shah:** Conceptualization (supporting); data curation (supporting); formal analysis (supporting); funding acquisition (supporting); investigation (supporting); methodology (supporting); project administration (supporting); resources (supporting); software (supporting); supervision (supporting); validation (supporting); visualization (supporting); writing – original draft (supporting); writing – review and editing (supporting). **Ashish Saxena:** Conceptualization (supporting); data curation (supporting); formal analysis (supporting); funding acquisition (supporting); investigation (supporting); methodology (supporting); project administration (supporting); resources (supporting); software (supporting); supervision (supporting); validation (supporting); visualization (supporting); writing – original draft (supporting); writing – review and editing (supporting). **Valerie F. Reyna:** Conceptualization (equal); data curation (supporting); formal analysis (supporting); funding acquisition (supporting); investigation (supporting); methodology (supporting); project administration (equal); resources (supporting); software (supporting); supervision (supporting); validation (supporting); visualization (supporting); writing – original draft (supporting); writing – review and editing (supporting). **Allyson Ocean:** Conceptualization (supporting); data curation (supporting); formal analysis (supporting); funding acquisition (supporting); investigation (supporting); methodology (supporting); project administration (supporting); resources (supporting); software (supporting); supervision (supporting); validation (supporting); visualization (supporting); writing – original draft (supporting); writing – review and editing (supporting). **Ronald Scheff:** Conceptualization (supporting); data curation (supporting); formal analysis (supporting); funding acquisition (supporting); investigation (supporting); methodology (supporting); project administration (supporting); resources (supporting); software (supporting); supervision (supporting); validation (supporting); visualization (supporting); writing – original draft (supporting); writing – review and editing (supporting). **Paul K. Maciejewski:** Conceptualization (supporting); data curation (supporting); formal analysis (supporting); funding acquisition (supporting); investigation (supporting); methodology (supporting); project administration (supporting); resources (supporting); software (supporting); supervision (supporting); validation (supporting); visualization (supporting); writing – original draft (supporting); writing – review and editing (supporting). **Andrew S Epstein:** Conceptualization (equal); data curation (equal); formal analysis (supporting); funding acquisition (equal); investigation (equal); methodology (equal); project administration (equal); resources (supporting); software (supporting); supervision (equal); validation (equal); visualization (equal); writing – original draft (supporting); writing – review and editing (supporting).

## FUNDING INFORMATION

This work was supported in part by a grant from the National Institute of Health/National Institute of Nursing Research (NIH/NINR) to Dr. Prigerson and Dr. Epstein (NR018693), a grant to Dr. Prigerson from the National Institute of Minority Health and Health Disparities (MD017704) and a grant from the National Cancer Institute (NCI) to Dr. Prigerson (CA197730).

## CONFLICT OF INTEREST STATEMENT

Dr. Epstein discloses royalties from Up‐To‐Date for peer reviewing GI medical oncology and palliative care topic reviews; Dr. Ocean discloses royalties from Guardant Health and Natera, Dr. Shah discloses research funding from Merck Inc., Bristol Meyers Squibb, and Oncoloys Biopharma; Dr. Derry‐Vick discloses an unrelated financial relationship with Dechra through her spouse's employment, Dr. Saxena discloses relationships to AstraZeneca Advisory Boards and G1 Therapeutics. All the remaining authors report nothing to disclose.

## CLINICALTRIALS.GOV IDENTIFIER

NCT04179305.

## Data Availability

https://endoflife.weill.cornell.edu/
